# Outcomes of breast conserving therapy: Recurrence, imaging findings and histological correlation

**DOI:** 10.4102/sajr.v27i1.2592

**Published:** 2023-04-20

**Authors:** Marara N. Sondezi, Ines Buccimazza, Ntombizakhona B. Madlala

**Affiliations:** 1 Department of Radiology, Nelson R. Mandela School of Medicine, University of KwaZulu-Natal, Durban, South Africa; 2 KwaZulu-Natal Breast Centre of Excellence, Department of Radiology, Inkosi Albert Luthuli Central Hospital, Durban, South Africa; 3 Department of Surgery, Nelson R. Mandela School of Clinical Medicine, University of KwaZulu-Natal, Durban, South Africa; 4 Breast and Endocrine Unit, Specialised Surgical Services, Inkosi Albert Luthuli Central Hospital, Durban, South Africa

**Keywords:** breast conserving surgery, breast conserving therapy, ipsilateral breast tumour recurrence, breast cancer recurrence, lumpectomy, wide local excision, quadrantectomy, breast mass, breast cancer, radiotherapy

## Abstract

**Background:**

Breast conserving therapy (BCT) is the mainstay therapy in patients with early breast cancer and selected patients with locally advanced breast cancer. No formal audit has been performed on BCT at our institution.

**Objectives:**

To determine the incidence and risk factors for ipsilateral breast tumour recurrence (IBTR). Study the imaging features of IBTR. Determine adherence to the proposed annual mammographic surveillance schedule.

**Method:**

Clinical, radiological and histopathological records of patients who underwent BCT from 01 January 2011 to 31 December 2015 were reviewed. Patients were followed up for at least 5 years.

**Results:**

Ninety-two patients were included in the study with a mean age of 54.3 years. Eighty of the 92 (87.0%) patients were imaged within 1-year post-BCT. Ipsilateral breast tumour recurrence was 6/92 (6.5%) with mean time to IBTR of 34.4 months. One of the 92 (1.0%) patients had a contralateral metachronous recurrence with no IBTR and 11/92 (12.0%) had distant metastases only. Pathological tumour size and extent (pT2) (68.5%) and pathological lymph node (pN0) (65.2%) were the most common locoregional staging. Infiltrating ductal carcinoma was the most common histological type (88%). Age < 35 years was associated with breast cancer recurrence (*p* < 0.01). Imaging findings of recurrence were microcalcification (odds ratio [OR]: 4), asymmetric density (OR: 4) and skin thickening (OR: 2.5).

**Conclusion:**

The occurrence of IBTR following BCT in our unit is acceptable and comparable to local and international units. The accuracy of assessing the post-BCT breast for IBTR is in keeping with international standards.

**Contribution:**

Improved radiological imaging interpretation of the post-BCT breast.

## Introduction

Breast conserving therapy (BCT) is the mainstay therapy and an alternative to mastectomy in patients with early breast cancer (EBC) and is the standard care at our centre, Inkosi Albert Luthuli Central Hospital.^1,2,3,4^ Breast conserving therapy offers the advantage of an acceptable cosmetic result; however, the post-treatment breast is difficult to manipulate for imaging due to patient pain threshold limitations and anatomical distortion that impairs breast compressibility, potentially masking an underlying ipsilateral breast tumour recurrence (IBTR).^5,6^ Early detection of IBTR is linked with greater survival outcomes.^7^ Clinically and radiologically diagnosed IBTR through surveillance screening in asymptomatic patients has a better prognosis than patient-detected recurrences.^7^ It is therefore imperative that the imaging findings are accurately assessed for prompt diagnosis and treatment of IBTR.^5,6,7^

The recurrence rates post-BCT vary with an acceptable range of 5% – 15%, although some studies have reported higher and lower incidences.^8,9,10,11,12,13^ Local studies performed at Groote Schuur Hospital in Cape Town showed a 5-year recurrence of 6.8% (median follow-up of 60 months)^14^ with a smaller study conducted in Johannesburg reporting a 5.5% recurrence (median follow-up of 65 months).^15^ Newer studies report significantly lower recurrence rates.^8^

Ipsilateral breast tumour recurrence leads to an increased risk for distant metastasis and is associated with decreased survival.^16,17^ The most frequent site is the original quadrant or the chest wall scar after radical surgery, which accounts for 60% – 95% of all cases.^16^

Several risk factors have been associated with IBTR. Young age (defined as either < 35 years or < 40 years) is associated with early onset and higher rates of IBTR when compared to older counterparts.^18,19^ A positive family history (particularly > 3 first-degree relatives) increases the risk of IBTR and contralateral recurrence.^20^ Breast cancer (BRCA) gene mutation 1 and 2 carriers are at risk of IBTR.^21^ Pathological margin status is an independent risk factor for IBTR.^22,23,24,25^ Triple negative subtype, ductal carcinoma *in situ* (DCIS) and an increased number of positive lymph nodes (particular N2) are associated with an increased risk for IBTR.^26,27^ Non-compliance with, interruptions and delays in initiating adjuvant radiotherapy and omission of a tumour bed boost are significantly associated with poorer outcomes.^12,28,29^ Tumours that fail to demonstrate pathological complete response after neoadjuvant chemotherapy are associated with a higher risk of IBTR.^27^

Ultrasonography is sensitive in distinguishing between post-operative complications and benign abnormalities but is inferior to mammography in detecting IBTR.^30^ Most IBTRs are described as hypoechoic lesions in contrast to benign lesions which tend to be anechoic or hyperechoic.^30^

The sensitivity of mammography in detecting IBTR in a post-treated breast ranges from 55% to 68%; this is due to surgery and radiation-induced changes in the parenchymal pattern.^5,17,31^ A small study by Pinksy demonstrated a 97% yield in diagnosing recurrences on mammography with 91% of cases being clinically occult and diagnosed solely on mammography.^32^ Mammographic characteristics of IBTR include calcifications at or near the tumour bed which usually resemble the primary tumour and may be pleomorphic, indistinct, coarse heterogeneous, or linear (75%); areas of architectural distortion (3%) and increasing skin oedema.^6,32^ Ipsilateral breast tumour recurrence may appear as a mass or asymmetric density of similar mammographic appearance to the primary tumour (*p* < 0.02).^33^

MRI is superior to ultrasound (US) and mammography due to its ability to identify additional disease in the ipsilateral and contralateral breast, detecting 15% and 10% of additional disease, respectively.^34,35^ In a study by Lehman et al., the sensitivity and specificity of MRI in the contralateral breast were 91% and 88% respectively with a negative predictive value of 99%.^36^ On mammographic and US images, tumour size was significantly underestimated by 14% and 18%, respectively (*p* < 0.005), while MR imaging showed no significant difference in size compared with that found at pathologic evaluation.^35^ In cases where mammography and/or ultrasonography are indeterminate for IBTR, MRI can reliably distinguish post-BCT scar tissue from IBTR when performed at least 12–18 months after completion of BCT, yielding a sensitivity of 90% – 100% and specificity of 83% – 93%.^5^ Pre-operative MRI also allows better assessment of the extent of a cancer in surgical naïve breasts, leading to better surgical planning (wider excision, extra biopsies and change of surgery from BCT to mastectomy); however, enhancement of benign lesions can lead to unnecessary wider surgery in 3% – 6% of patients.^37^

At our institution, BCT is the preferred treatment method for managing EBC and selected patients with locally advanced breast cancer. Ultrasonography, mammography and MRI in selected cases are performed at 6 months post-BCT at a time where post-surgical changes may mimic IBTR. We investigated the outcomes and imaging characteristics post-BCT, correlating with histopathological reports.

## Method

This was a qualitative, retrospective study of all consecutive adult women (over 18 years) who underwent BCT for EBC and selected cases of locally advanced breast cancer at a specialised breast unit in Inkosi Albert Luthuli Central Hospital over the period 01 January 2011 – 31 December 2015 and who were followed up for at least 5 years post-surgery to determine recurrence. All patients must have undergone US, mammography and/or MRI studies during the follow-up period. Patients with benign pathological diagnoses, incomplete histopathological reports, those lost to follow-up or not imaged post-BCT and male patients with breast cancer were excluded.

Clinical, chemotherapy treatment, radiological and histopathological records for patients who underwent BCT for breast cancer during the study period were collected. Relevant patient data collected by means of a data sheet included age, family history, history of previous BCT, co-morbidities, clinical assessment (tumour size) and radiological features of the primary tumour as well as the recurrent tumour (size, quadrant, calcifications, mass, distortion, skin thickening, presence of axillary lymph nodes, contralateral breast lesion, distant lesion and enhancement) with respect to US, mammography and/or MRI. Data on patient’s radiotherapy treatment were also collected. Histopathological reports of patients who had imaging features which were suspicious for recurrence and had biopsies, were studied. The time, location, imaging features and management of recurrences were collected.

The datasheet consisting of a set of matrices was completed. Each patient was assigned a number on their respective datasheet to ensure patient confidentiality. The radiological images were reviewed and interpreted by two practising radiologists (a qualified specialist and a radiologist in training) and findings recorded on the datasheet. The radiologists were not blinded to one another’s assessment. The outcome event (death, disease recurrence, survival) was recorded from the time of surgical intervention to the end of the follow-up period. These data were captured in Microsoft Excel.

### Data analysis

Descriptive statistics were used to summarise the data. Frequencies and percentages were used for categorical data. Frequency distributions of numeric variables were examined for normality, means (standard deviation [s.d.]) and median (interquartile range [IQR]). Risk factors associated with recurrence (Yes/No) were examined using Chi-square tests for categorical data and *T*-test or Mann–Whitney for numeric and ordinal variables. Odds ratios (ORs), 95% confidence intervals (CIs) and *p*-values were reported. Significant *p*-value was set at *p* < 0.05.

Sensitivity and specificity of imaging findings were studied. A multivariable logistic regression was used to identify independent risk factors in patients with recurrence. Data were analysed in Stata^®^ version 15.1 statistical software.

### Ethical considerations

Approval for this study was obtained from the University of KwaZulu-Natal Biomedical Research Ethics Committee with protocol reference number BREC/00002843/2021. All patient identifiers were concealed to ensure patient anonymity.

## Results

### Patient and disease characteristics

A total of 143 patients underwent BCT during the study period. Fifty-one patients were excluded from the study either because they were lost to follow-up (*n* = 50) or had benign pathology (*n* = 1). Therefore, 92 patients who underwent BCT during the study period were included (Table 1). The mean age was 54.3 years (range: 31–79 years). Most patients were between the ages of 51–79 years (60.9%, 56 patients). The prevalence of HIV in the study population was 8.7% (eight patients) with diabetes mellitus affecting 23.9% (*n* = 22) of patients. The relationship between co-morbid diseases (HIV and diabetes mellitus) was not statistically significant (*p* = 0.21).

**TABLE 1:** Patient demographics comparing patients with and without ipsilateral breast cancer recurrence.

Variable	Women with ipsilateral breast cancer recurrence (*n* = 6)		Women without ipsilateral breast cancer recurrence (*n* = 86)		Total (*N* = 92)	*p* †
	*n*	%	*n*	%		
**Age (years)**						
≤ 35	4	57.1	3	42.9	7	< 0.001
35-50	1	3.4	28	96.6	29	-
≥ 51	1	1.8	55	98.2	56	-
**Co-morbid disease**						
HIV	1	12.5	7	87.5	8	0.21
Diabetes	0	0.0	22	100.0	22	-
None	5	8.1	57	91.9	62	-
**BRCA gene**						
BRCA 1 positive	0	0.0	1	100.0	1	1.0
BRCA 2 positive	0	0.0	2	100.0	2	-
Not stated	6	6.7	83	93.3	89	-
**Family history**						
< 3 First-degree relative	2	16.7	10	83.3	12	0.036
> 3 First-degree relatives	1	33.3	2	66.7	3	-
No family history	3	3.9	74	96.1	77	-

Note: Demographics of 92 patients who underwent BCT.

BRCA, breast cancer; BCT, breast conserving therapy.

† , Statistical test – Fisher’s exact.

### Incidence of recurrence

During the median follow-up period of 70.3 months (range: 1–135.1 months), a total of six patients (6.5%) of the 92 patients who underwent BCT developed IBTR during the study period. The mean time to IBTR was 34.4 months (*p* = 0.01). The histological subtype of the IBTR was invasive ductal carcinoma in all cases. Four (66.7%) of the six patients with IBTR also presented with distant metastasis. Incidentally, over the study period, one patient presented with a contralateral metachronous DCIS at 67 months and no IBTR, and 11 (12.0%) patients presented with metastatic recurrence only without the presence of IBTR. The frequency of metastatic recurrence sites was bone 56.3%, lung 43.8%, pleura 31.3%, liver 31.3%, axillary lymph nodes 18.8% and brain 12.5%. One patient with IBTR and bone metastasis died during the study period at 13 months. The combined mean time to breast or metastatic recurrence was 32.2 months (*p* < 0.01).

### Risk factors for recurrence

Age < 35 years was significantly associated with IBTR (*p* < 0.001). Three patients tested positive for a BRCA gene mutation: one patient having BRCA 1 mutation and two demonstrating a BRCA 2 mutation. Two of the patients with BRCA mutation presented with distant metastasis with no evidence of IBTR. The remaining patient had neither IBTR nor distant metastasis. Fifteen patients had a positive family history of breast cancer. Of these, 80% (12/15 patients) had fewer than three first-degree relatives with breast cancer and 20% (three patients) had more than three first-degree relatives with breast cancer. Of the six patients with IBTR, three had a family history of breast cancer: two had fewer than three first-degree relatives with breast cancer and one had more than three first-degree relatives with breast cancer (*p* = 0.036).

### Pathological factors

The majority of patients had pathological tumour size and extent T2 and pathological lymph node N0 staging correlating to stage II with a mean tumour size of 3.3 cm (range 1.0 cm – 5.0 cm) (Table 2). Pathological T2 lesions accounted for 68.5% (63/92 patients) of cases with Tis, T1 and T3 lesions representing 3.3% (3/92 patients), 17.5% (16/92 patients) and 10.9% (10/92 patients) of cases, respectively. Pathological T2 staging did not demonstrate statistically significant correlation with IBTR (6.3%, *p* = 1.00). The majority of patients had N0 nodal staging (65.2%, 60 patients). Nodal staging was not associated with IBTR (*p* = 0.18).

**TABLE 2:** Demonstrating the influence of pathological status on recurrence.

Variable	No. of patients with ipsilateral breast tumour recurrence (*n* = 6)		No. of patients without ipsilateral breast tumour recurrence (*n* = 86)		Total	*p*
	*n*	%	*n*	%		
**Pathological tumour size (pT)**						
pTis	0	0.0	3	100.0	3	0.1*
pT1	2	12.5	14	87.5	16	-
pT2	4	6.3	59	93.6	63	-
pT3	0	0.0	10	100.0	10	-
**Pathological nodal size (pN)**						
pN0	2	3.3	58	96.6	60	0.18*
pN1	4	12.5	28	87.5	32	-
**Histological subtype**						
Invasive ductal carcinoma	6	100.0	74	92.5	80	0.9*
Ductal carcinoma *in situ*	0	0.0	7	100.0	7	-
Other	0	0.0	5	100.0	5	-
Invasive papillary	0	0.0	1	100.0	1	-
Medullary	0	0.0	1	100.0	1	-
Neuroendocrine differentiation	0	0.0	1	100.0	1	-
Papillary	0	0.0	1	100.0	1	-
Undifferentiated	0	0.0	1	100.0	1	-
**Tumour grade**						
Grade I (low)	0	0.0	9	100.0	9	0.5**
Grade II (intermediate)	4	8.2	45	91.8	49	-
Grade III (high)	2	9.1	20	90.9	22	-
Not stated	0	0.0	12	100.0	12	-
**Receptor status**						
ER/PR positive	5	6.6	71	93.4	76	1*
ER/PR negative	1	6.2	15	93.8	16	-
**Margin status**						
Negative	5	5.7	83	94.3	88	0.24*
Positive	1	25.0	3	75.0	4	-
**Lymphovascular invasion**						
Negative	3	4.2	68	95.8	71	0.08*
Positive	3	17.6	14	82.4	17	-

ER/PR, estrogen receptor/progesterone receptor.

* , Fisher’s exact;

** , Two-sample Wilcoxon rank-sum.

Invasive ductal carcinoma represented 86.9% (80 patients) of the cancers while DCIS represented 7.6% (seven patients). The other histological subtypes included medullary, papillary, neuroendocrine and undifferentiated cancers. The relationship between IBTR and histological subtype was not statistically significant in this study (*p* = 0.9).

Estrogen receptor/progesterone receptor (ER/PR) positive Human epidermal growth factor receptor 2 (HER-2) negative was the most commonly expressed hormonal status (61.9%, 57 patients). The risk of IBTR was not associated with hormone receptor positive status (*p* = 1.00). Grade 1, 2 and 3 tumours represented 9.7%, 53.2% and 23.9%, respectively. Histological tumour grade was not associated with IBTR (*p* = 0.5). Seventeen (18.5%) patients had lymphovascular invasion with 10 (10.9%) patients demonstrating cancers with perineural invasion. Three of the patients with IBTR also had lymphovascular invasion. The relationship between lymphovascular invasion and recurrence was not scientifically significant (17.6%, *p* = 0.08). In 63 patients, the lymph nodes were described as clinically suspicious of metastasis; however, only 32 were proven to be pathological on histology. Of these pathological lymph nodes, 10 patients had extranodal extension. Three patients with IBTR had pathological lymph nodes which demonstrated extranodal extension; however, the correlation between IBTR and extranodal extension was not statistically significant (*p* = 0.42).

Of the 92 patients who underwent BCT, three (4.3%) had involved margins with DCIS after the initial excision. Two of the three patients with involved margins had grade 2 disease and the remaining patient had grade 3 disease. In respect of the management of positive margins, two patients underwent total mastectomy and the remaining patient had a re-excision. No residual tumour was noted in the re-excision specimen. None of these patients developed IBTR or distant metastasis. A positive margin status did not confer statistically significant results (25%, *p* = 0.24).

### Chemoradiotherapy treatment

A total of 36 patients (39.1%) were offered neoadjuvant chemotherapy. Of these, 23 (63.9%) achieved complete clinical response. Five (5, 13.9%) had an incomplete clinical response while eight (22.2%) demonstrated no response to chemotherapy. The most common chemotherapy regimen administered was a 5-Fluorouracil, Epirubicin, Cyclophosphamide combination. Half (3/6) of the patients with IBTR had neoadjuvant chemotherapy and demonstrated incomplete or no clinical response. The correlation between pathological complete response post neoadjuvant chemotherapy and the development of IBTR was not statistically significant (*p* = 0.6454). Forty-eight (52.2%) of patients received adjuvant chemotherapy including the six patients with IBTR. Four (66.6%) of the patients with IBTR received 5-Fluorouracil, Epirubicin, Cyclophosphamide combination while the remaining patients received docetaxel. Seventy-seven (77.2%) of the 92 patients received endocrine therapy, most commonly tamoxifen.

A total of 82 patients (89.1%) completed radiotherapy, inclusive of the six patients with IBTR. Reasons for the remaining 10 patients not receiving radiotherapy included: mastectomy for EBC (2/10), overweight for the radiotherapy machine (1/10), development of metastasis before initiating radiotherapy (1/10), no record of radiotherapy (6/10).

Of the 82 patients who completed radiotherapy, 44 patients (53.6%) experienced delays in initiating radiotherapy, either due to the compromised radiotherapy service as a result of oncologist shortage during the study period, public worker industrial action, patient confusion with radiotherapy dates or patient financial constraints. Half of the patients with IBTR (3/6 patients) and 47.6% (41/86 patients) without IBTR, experienced delays initiating radiotherapy. However, the correlation between radiotherapy delay and the development of recurrence was not statistically significant (*p* = 0.9). Five patients (6.1%) experienced interruption in radiotherapy due to skin reactions. None of the patients with IBTR had interruption of radiotherapy. The relationship between interruption in radiotherapy and recurrence was not statistically significant (*p* = 0.3).

All of the patients with IBTR received radiotherapy and a tumour bed boost. The average dose given was 46.5 Gy with a range of 40.5 Gy – 70 Gy. A tumour bed boost was given to only 77/82 patients (93.9%). Of the remaining patients, five patients (6.1%) did not receive radiotherapy tumour boost due to resource limitation during the study period (2/5) and three patients had no record of tumour boost in their files.

### Post-operative imaging findings

**FIGURE 1:**
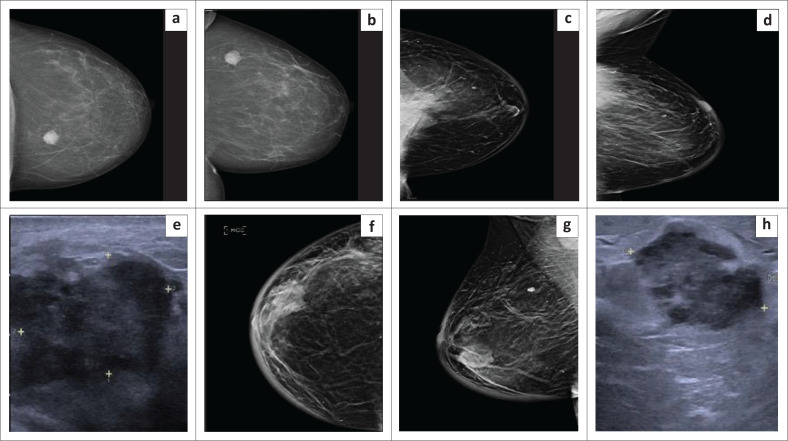
Mammogram and US images of a 34-year-old female with a strong family history of breast cancer. Images (a) and (b) demonstrate the pre-treatment mammogram craniocaudal (CC) and mediolateral (MLO) views. In the left breast, there is a solitary hyperdense spiculated mass which correlated to a grade III triple negative invasive ductal carcinoma (IDC). Five years post-breast conserving therapy, the patient presented with ipsilateral breast tumour recurrence as demonstrated by images (c–e). Images (c) and (d), CC and MLO mammograph demonstrating a lobulated hyperdense mass in the upper outer quadrant of the left breast. The mass extends into the retromammary space with tethering of the pectoralis muscle suggestive of chest wall invasion. Intralesional pleomorphic calcifications noted. On US (image e), there is a lobulated, hypoechoic, solid non-parallel mass as demonstrated by the yellow callipers. Palliative chemotherapy was prescribed due to metastatic disease. A year later, the patient presented with a new right breast mass. Images (f) and (g) are CC and MLO mammographs which demonstrate a hyperdense irregular lobulated mass in the outer lower quadrant of the right breast with associated pleomorphic calcifications; associated skin thickening is noted. Histology confirmed pathological right axillary lymph nodes. Note the coarse calcification in the upper quadrant of the right breast which is benign in nature. On the adjoining US image (h), there is an irregular lobulated hypoechoic non-parallel soft tissue mass denoted by the yellow callipers. Histology confirmed IDC in keeping with radiological evidence of contralateral metachronous breast recurrence.

Only 80/92 (87.0%) patients were imaged within 1-year post-BCT (Table 3). Of these, only 22 patients (27.5%) were imaged by both US and mammogram. Fifty-two patients (65%) had only a mammogram, four (5%) only US and two (2.5%) only MRI in the first post-BCT imaging series. Based on the post-BCT images, a total of 32 patients (40%) underwent biopsy to confirm radiological suspicion of recurrence. In seven of these patients the biopsy returned a positive diagnosis for malignancy; six IBTR and one contralateral metachronous cancer (Figure 1).

**TABLE 3:** Mammographic features of ipsilateral breast tumour recurrence.

Histological proven IBTR – mammographic features	Yes (*n* = 6)		No (*n* = 26)		Total (*N* = 32)	*p*	OR	95% CI
	*n*	%	*n*	%				
**Skin thickening**						0.47	2.5	0.18–34.67
Absent	4	16.7	20	83.3	24	-	-	-
Present	1	33.3	2	66.7	3	-	-	-
Not stated	1	20.0	4	80.0	5	-	-	-
**Degree of skin thickening (> 3 mm)**						0.9	-	-
Absent	5	19.2	21	80.8	26	-	-	-
Present	0	0.0	1	100.0	1	-	-	-
Not stated	1	20.0	4	80.0	5	-	-	-
**Locoregional lymph nodes**						-	-	-
Absent	5	18.5	22	81.5	27	-	-	-
Present	0	0.0	0	0.0	0	-	-	-
Not stated	1	20.0	4	80.0	5	-	-	-
**Asymmetric density or mass**						0.25	4	0.38–41.74
Absent	1	8.3	11	91.7	12	-	-	-
Present	4	26.7	11	73.3	15	-	-	-
Not stated	1	20.0	4	80.0	5	-	-	-
**Increasing oedema**						0.9	-	-
Absent	5	21.7	18	78.3	23	-	-	-
Present	0	0.0	3	100.0	3	-	-	-
Not stated	1	16.7	5	83.3	6	-	-	-
**Calcifications**						0.3	4	0.53–30.16
Absent	2	11.1	16	88.9	18	-	-	-
Present	3	33.3	6	66.7	9	-	-	-
Not stated	1	20.0	4	80.0	5	-	-	-
**Types of calcifications**						-	-	-
Pleomorphic	3	100.0	0	0.0	3	-	-	-
Indistinct	0	0.0	2	100.0	2	-	-	-
Coarse heterogenous	0	0.0	4	100.0	4	-	-	-

Note: Imaging features of recurrence on mammogram of 32 patients whose mammograms were suspicious of recurrence.

IBTR, ipsilateral breast tumour recurrence; OR, odds ratio; CI, confidence interval.

**FIGURE 2:**
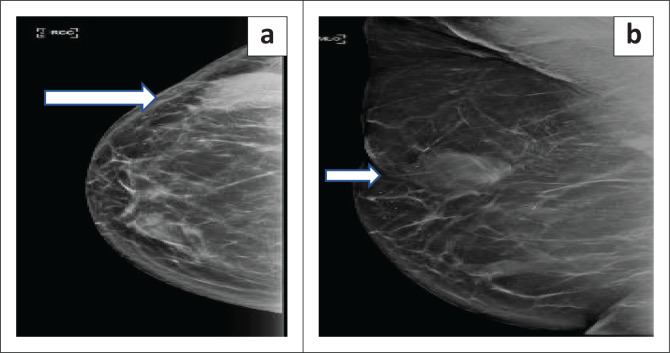
Images from a 51-year-old woman with previous right breast estrogen receptor positive grade II invasive ductal carcinoma (IDC) treated with breast conserving therapy. On surveillance, (a) craniocaudal (CC) and (b) mediolateral (MLO) mammogram views of the right breast demonstrate a hyperdense mass in the outer quadrant of the right breast with partially obscured margins. The MLO view better depicts associated pleomorphic microcalcifications. Biopsy confirmed ipsilateral breast tumour recurrence.

**FIGURE 3:**
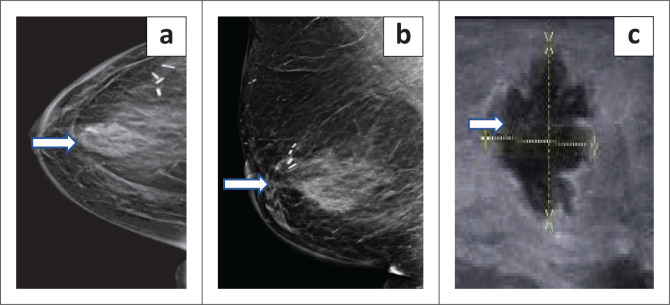
These are images of a 66-year-old female with previous left breast carcinoma triple positive invasive ductal carcinoma (IDC) grade 2 who underwent BCT with contralateral symmetrisation. Subsequently, this patient developed metachronous right breast carcinoma which was treated with WLE as per staples in the (a) craniocaudal and (b) mediolateral mammogram views. A year later, the patient developed ipsilateral breast tumour recurrence in the right breast which is demonstrated by the arrow in (a) and (b) indicating an irregular, isodense lesion in the central right breast within the middle segment. The posterior margin is obscured while the anterior margin demonstrates microlobulations; no sinister calcifications; minimal skin thickening, no nipple retraction. (c) Greyscale ultrasound image which demonstrates a non-parallel irregular microlobulated hypoechoic solid mass in the right breast (marked by yellow calipers) with surrounding oedema. This mass was proven on histology as estrogen receptor/progesterone receptor positive high grade ductal carcinoma in situ.

**FIGURE 4:**
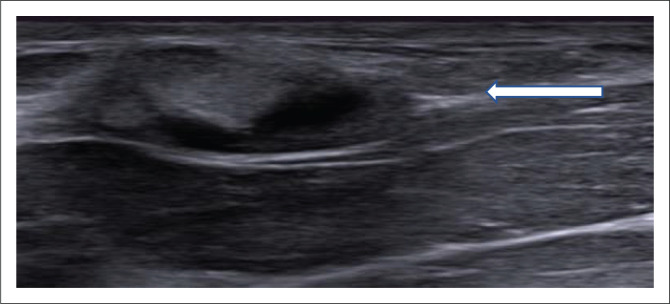
Greyscale ultrasound image of a 50-year-old female initially diagnosed with estrogen receptor/progesterone receptor+ invasive ductal carcinoma (IDC), grade II with an intraductal component. Four months post-BCT, the patient developed a mass. The ultrasound image shows an anechoic parallel mass with a non-dependant soft tissue component and mixed posterior acoustic change. The corresponding mammogram showed a soft tissue mass. The final BIRADS classification was 4B and the patient underwent stereotactic biopsy which confirmed fat necrosis.

**FIGURE 5:**
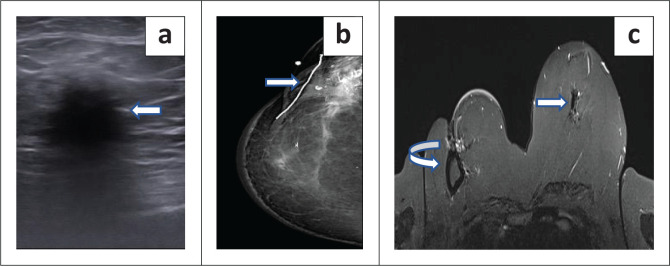
Images of 62-years-old female (a) Grayscale ultrasound image demonstrates an ill-defined, anechoic lesion with posterior acoustic shadowing (arrow). (b) The corresponding craniocaudal view demonstrates coarse calcifications at the surgical bed (arrow). (c) Dynamic T1 post gadolinium axial breast MRI demonstrates a rounded, well-defined lesion of low intensity with no post contrast enhancement in the right upper outer breast (curved arrow) which correlated with the coarse calcification on the mammogram. The area of calcification in the central left breast (straight arrow) correlated to grouped coarse calcifications. The right breast was reported as fat necrosis (benign lesion [BIRADS 2]) and the left suspicious calcifications (high likelihood of being cancer [BIRADS 4C]) warranting biopsy. Histology of the left breast proved fat necrosis.

An asymmetrical density or mass was found in 46.9% (15/32) patients who underwent biopsy (*p* < 0.001). Of these patients, asymmetric density was found in four patients with IBTR and 11 patients without IBTR with an OR of 4 (95% CI: 0.38–41.74, *p* = 0.25). Increasing breast oedema was observed in 9.4% (3/32) patients who underwent biopsy (*p* = 0.03). None of the patients who had post-operative increasing breast oedema had an IBTR (*p* = 0.9). Calcifications were reported in nine patients (28.1%) who underwent biopsy: coarse (four), pleomorphic (three), indistinct (two) (Figure 2 and Figure 3). All patients with pleomorphic calcifications had biopsy-proven recurrence. None of the patients with coarse and indistinct calcifications developed recurrence. The presence of calcifications was associated with IBTR with OR of 4 (95% CI: 0.53–30.16, *p* = 0.3). Skin thickening (> 3 mm) was described in three patients who were biopsied. Recurrence occurred in one patient who had skin thickening (> 3 mm) with OR of 2.5 (95% CI: 0.18–34.67, *p* = 0.47). This patient also had a breast mass on mammogram in addition to skin thickening. Of the 25 histologically negative biopsies, fat necrosis accounted for 18 while four represented normal fibrofatty breast tissue, one fibroadenoma, and one each for fibrous tissue and blood (Figure 4 and Figure 5). Benign post-operative changes were indistinct or coarse calcifications, absence of lymphadenopathy and absence of skin thickening (> 3 mm).

A comparison between mammographic findings of IBTR and the histopathological reports was made. In this cohort, we found that the sensitivity and specificity of predicting breast cancer recurrence on mammography were 100% and 70.6% respectively with a positive and negative predictive value of 21.9% and 100%, respectively.

**FIGURE 6:**
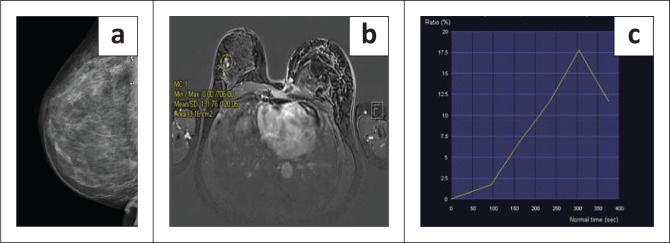
Images of a 37-year-old female post-breast conserving therapy for right breast cancer. Two years post-BCT, the patient developed a mass in the surgical bed. (a) Mediolateral mammogram demonstrates an isodense mass with pleomorphic calcifications within the surgical bed. (b) T1 weighted post gadolinium fat saturated axial breast MRI demonstrates nodular mass-like enhancement in the upper outer quadrant of the right breast encircled by the yellow region of interest. The corresponding enhancement kinetic curve (c) corresponds to a type 3 dynamic curve, consistent with a malignant lesion. Histology confirmed moderately differentiated invasive ductal carcinoma.

MRI was utilised to complement US or mammogram in six additional patients due to dense breasts. Collectively, MRI was used in eight patients during the first round of post-operative imaging which resulted in two positive diagnoses of breast recurrence (Figure 6).

## Discussion

This study was performed to review outcomes following BCT in a single tertiary centre. Nearly all the tumours were pT1 and pT2 lesions in keeping with international standards and guidelines for BCT in EBC.^3,38^ This study determined a relative IBTR of 6.5% with a mean time to recurrence time of 34.4 months and is comparable to local and international recurrence rates which are reported as acceptable if between the 5% – 15% range.^1,11,14,16,19^

Age < 35 years was strongly associated with IBTR in our cohort, in keeping with published literature. The margin positivity was 3.3%; however, none of these patients with positive margins developed IBTR. Although margin status is associated with a higher IBTR, we could not demonstrate this relationship in the current study possibly due to early re-excision which mitigated the recurrence risk and the small number of patients in our series.

Absence of pathological complete response to neoadjuvant chemotherapy is associated with increased IBTR; however, in this study this correlation was not significant.^27^ Radiotherapy is known to be protective against IBTR.^39,40,41,42,43^ However, only 82/92 (89.1%) patients completed radiotherapy in this study. Contrary to current literature, we could not demonstrate the relationship between omission of radiotherapy and recurrence (*p* = 0.9). In the current study, the correlation between interruption and delays in radiotherapy and IBTR was not statistically significant (*p* = 0.3). Kolasińska et al. found that IBTR was the first treatment failure in patients who do not receive a tumour boost, hazard ratio (HR) 0.65 (99% CI: 0.52–0.81, *p* < 0.0001); however, this relationship could not be demonstrated in this study.^12^

The evaluation of the post-BCT breast is difficult due to overlapping features between post-surgical changes and recurrent malignancy.^5,6^ Imaging features that were associated with recurrence in our series are similar to those reported in the literature, and included an enlarging mass, microcalcifications, increased skin thickening (> 3 mm), increasing oedema and asymmetric density.^5,6^ In the current study, asymmetric density and microcalcifications most commonly correlated with recurrence, followed by skin thickening. The lack of statistical significance may be due to the small sample size. Contrary to current literature, we could not demonstrate a relationship between linear, coarse and indistinct calcifications and recurrence. In this study, only pleomorphic calcifications were seen with recurrence.

The sensitivity and specificity of predicting breast cancer recurrence on mammography are 100% and 70.6% respectively with a positive and negative predictive value of 21.9% and 100%, respectively. This sensitivity is comparable and slightly better than international standards which describe a sensitivity on mammography of 55% – 68%.^5,44,45^ We anticipate that the use of contrast enhanced mammogram at our institution will assist in improving diagnostic imaging accuracy.^46^ Features which correlated with benign breast changes included absence of skin thickening (> 3 mm), indistinct and coarse calcifications, absence of lymphadenopathy.

The study also revealed a lack of adherence to a standard post-treatment surveillance protocol. Of the 80 patients imaged within 1-year post-BCT, only 22 (27.5%) were imaged by both US and mammogram. Factors influencing poor adherence to imaging protocol include patient factors (financial constraints) and non-functional mammogram machines at the base hospitals. It is therefore imperative to ensure adequate access to imaging during the surveillance period to adequately assess the breast.

### Limitations

This study has a number of limitations due to the retrospective nature of the study. It was a single centre study, where most patients presented with locally advanced disease; therefore, the number of patients suitable for BCT was small. This number was further reduced by patients lost to follow-up during transfer to off-site oncology centres. These factors contributed to the small sample size.

There was limited access to the adjuvant treatments as patient files were transferred to the off-site oncology follow-up facility site. Where files were available, some information was incomplete due to poor record keeping and non-standardisation of patient records which is a general challenge in the public sector.

## Conclusion

The study demonstrates that BCT outcomes at our centre are within local and international standards in terms of margin positivity and recurrence rate. The accuracy of assessing the post-BCT breast for recurrences is in keeping with international standards. The sensitivity and specificity of diagnosing recurrence on mammogram surpasses current international standards. Although the correlation between delay and interruption of radiotherapy to IBTR was not statistically significant in this study, it has highlighted patient and systemic barriers to healthcare. Implementation of efficient decentralised breast imaging centres could improve patient adherence and timeous annual breast imaging following breast cancer therapy.
